# Opioids drive breast cancer metastasis through the δ-opioid receptor and oncogenic STAT3

**DOI:** 10.1016/j.neo.2020.12.011

**Published:** 2021-01-16

**Authors:** Sabrina Tripolt, Heidi A. Neubauer, Vanessa M. Knab, Dominik P. Elmer, Fritz Aberger, Richard Moriggl, Daniela A. Fux

**Affiliations:** a Institute of Pharmacology and Toxicology, University of Veterinary Medicine Vienna, Vienna, Austria; b Institute of Animal Breeding and Genetics, University of Veterinary Medicine Vienna, Vienna, Austria; c Department of Biosciences, Cancer Cluster Salzburg, Paris-Lodron University of Salzburg, Salzburg, Austria

**Keywords:** Breast cancer, STAT3, Opioid, Metastasis, EMT, OR, Opioid receptor, DOR, OPRD1, δ (delta)-opioid receptor, EMT, Epithelial–mesenchymal transition, GPCR, G protein-coupled receptors, JAK1/2, Janus kinase 1/2, STAT3, Signal transducer and activator of transcription 3

## Abstract

The opioid crisis of pain medication bears risks from addiction to cancer progression, but little experimental evidence exists. Expression of δ-opioid receptors (DORs) correlates with poor prognosis for breast cancer patients, but mechanistic insights into oncogenic signaling mechanisms of opioid-triggered cancer progression are lacking. We show that orthotopic transplant models using human or murine breast cancer cells displayed enhanced metastasis upon opioid-induced DOR stimulation. Interestingly, opioid-exposed breast cancer cells showed enhanced migration and strong STAT3 activation, which was efficiently blocked by a DOR-antagonist. Furthermore, opioid treatment resulted in down-regulation of E-Cadherin and increased expression of epithelial-mesenchymal transition markers. Notably, STAT3 knockdown or upstream inhibition through the JAK1/2 kinase inhibitor ruxolitinib prevented opioid-induced breast cancer cell metastasis and migration *in vitro* and *in vivo*. We conclude on a novel mechanism whereby opioid-triggered breast cancer metastasis occurs via oncogenic JAK1/2-STAT3 signaling to promote epithelial-mesenchymal transition. These findings emphasize the importance of selective and restricted opioid use, as well as the need for safer pain medication that does not activate these oncogenic pathways.

## Introduction

Opioids are potent analgesic drugs that are indispensable in cancer therapy, as their use alleviates pain during and after tumor resection surgery. Moreover, opioids are applied to relieve cancer-related pain resulting from the tumor pressing on different organs or causing massive inflammation/tissue damage. Opioids are also administered to manage pain arising from chemo- or radiotherapy [1]. However, opioids are widely misused and often given to risk group patients of older age with chronic disease/pain, despite that they are more susceptible to develop invasive cancers.

The use of opioids in cancer patients is a controversial debate. Epidemiologic studies showed that the risk of cancer recurrence and metastasis is enhanced when surgical tumor removal was performed under general anesthesia with systemic opioid administration [2], [3], [4]. Furthermore, survival probabilities of tumor patients were found to be reduced by using opioid-based general anesthesia [5], [6], [7]. Enhanced metastasis was also reported in opioid-treated rodents with mammary carcinomas [8,9], but the cellular and molecular mechanisms underlying these observations remain unclear. It is, therefore, important to investigate whether these observed protumorigenic actions are opioid-specific effects involving specific downstream signaling mechanisms.

The classical analgesic effect of opioids is mediated by µ-, δ-, and κ-opioid receptors, which belong to the family of G protein-coupled receptors (GPCRs). Opioid receptors are linked to different intracellular signaling cascades including ERK1/2 and AKT signaling [10], which contribute to various cellular processes including proliferation, differentiation, and survival [11,12]. Opioid receptors are most strongly expressed in neurons of the nociceptive system, but can also be found in heart, immune system, gastrointestinal tract and reproductive system cells [13,14]. Recently, high expression of δ-opioid receptors (DORs) was observed in tissue samples from breast cancer patients, which correlated with tumor progression and poor prognosis [15]. Given that breast cancer patients often undergo significant surgical procedures for axial lymph node clearance and mastectomy, requiring general anasthesia, we investigated the role of opioid-mediated DOR signaling on the migration and metastasis of mammary tumor cells.

Here, we reveal through comprehensive data mining that DOR mRNA is significantly overexpressed in various independent cohorts of breast cancer samples, as well as other solid cancer types, from patients with increased cancer progression or metastatic incidence. We employed orthotopic transplant models using human and murine breast cancer cell lines to demonstrate that opioid-induced DOR stimulation enhances metastasis. Importantly, we explored the mechanism of how DOR activation promotes breast cancer progression, finding strong induction of oncogenic JAK1/2-STAT3 signaling and epithelial-mesenchymal transition (EMT) markers. Notably, we utilized pharmacologic inhibition of JAK1/2 and stable genetic STAT3 knockdown to conclude that opioid-triggered STAT3 activation promotes breast cancer migration and metastasis. Together, these findings reveal novel mechanistic insights into opioid-mediated breast cancer progression and dissemination.

## Materials and methods

### Cell culture

Breast cancer cell lines MDA-MB-231, MCF-7, T47D, 4T1, and human glioblastoma cells LN299 were purchased from the American Type Culture Collection (ATCC; VA, USA). LN299 and human breast cancer cell lines were maintained in Dulbecco´s modified Eagle´s medium (DMEM; Sigma Aldrich, MO, USA) supplemented with 10% fetal bovine serum (FBS; PAA Laboratories Inc., AUT) and 100 U/mL Penicillin-Streptomycin. 4T1 cells were cultured in RPMI-1640 medium supplemented with 10% FCS and 100 U/mL Penicillin-Streptomycin. Cells were maintained at 37°C with 5% CO_2_.

### RNA interference and lentiviral transduction

Cells with stable *STAT3* knockdown were generated by using lentiviral RNA interference as described in Kasper et al using Metafectene pro (Biontex, GER) transfection reagent. Viral particles were produced in transfected 293FT cells for each of the following short hairpin RNA (shRNA) constructs (Mission TRC shRNA library, Sigma Aldrich): control shRNA (SHC002), shRNA *STAT3* (shSTAT3) #1 (TRCN0000071456) and #2 (TRCN0000020843). Lentiviral transduction of MCF-7, MDA-MB-231, and T47D cells with *control* and *shSTAT3* constructs was performed as previously described [16]. Two days after transduction, breast cancer cells were selected with Puromycin and further analyzed for knockdown efficiency by Western blot and qPCR.

### Cell extracts and immunoblotting

Immunoblotting was performed as previously described [17]. Briefly, cells were treated with 1 µM [D-Ala^2^, D-Leu^5^]-Enkephalin acetate salt (DADLE; Bachem, CH; in text indicated as opioid) or rhIL-6 (200 ng/mL; 20 min), rhIL-2 (15 min; 100 ng/mL;), rhEGF (5 min; 100 ng/mL) and rhTGF (40 min; 1 ng/mL) as indicated and lysed with ice-cold RIPA buffer (10 mM Tris-Cl (pH 8.0), 1 mM EDTA, 0.5 mM EGTA, 1% Triton X-100, 0.1% sodium deoxycholate, 0.1% SDS, 140 mM NaCl). Total cell lysates (30 µg) were subjected to 7% SDS polyacrylamide gel electrophoresis and blotted on nitrocellulose membrane (GE Healthcare, GBR). Membranes were blocked with 5% BSA in TBS/Tween-20 (0.1%) and probed with antibodies against ACTIVE-Β-CATENIN (ABC) (Cell Signaling Technology (CST), MA, USA; 95625), E-CADHERIN (14472), GAPDH (2118), phospho-p42/p44 (Thr202/Tyr204) (9101), total SMAD2/3 (8685), phospho-SMAD2 (Ser465/467)/SMAD3 (Ser423/425) (8828), SNAIL (3879), phospho-STAT3 (Tyr705) (9131), total p42/p44 (9102), total STAT3 (12640), total STAT5 (9363), phospho-STAT5 (Tyr694) (Becton Dickinson (BD), NJ, USA; 611964), DOR-1 (Santa Cruz Biotechnology (SCB), TX, USA; 9111), HSC-70 (7298), TWIST (81417) or β-ACTIN (69879). After washing and membrane incubation with respective secondary antibodies, proteins were detected by chemiluminescence using LumiGLO Reagent (CST) and ChemiDoc XRS+ (Bio-Rad, CA, USA). The original uncropped Western blot images with molecular weight ladders visible are provided as a Supplemental Information file.

### Flow cytometry analysis

To measure proliferation, 2 × 10^4^ cells were seeded in 6-well plates, treated with 1 µM opioid, and counted by FACS each day for 4 d. For cell cycle analysis, cells were treated with 1 µM opioid for 24 h and subjected to cell cycle analysis by means of propidium iodide staining as previously reported [18]. Cell staining was recorded by a BD FACS Canto II flow cytometer and FACS Diva software (BD) as described [17].

### Cell migration

Migration of breast cancer cells was assessed by scratch assay. Specifically, cells were seeded onto 6-well plates at 5 × 10^4^ cells/well. Confluent monolayers were scratched with a sterile 1 mL tip and washed 2 times with PBS. Cells were maintained in fresh DMEM medium and exposed to 1 µM opioid, 10 µM naltrindole (selective DOR antagonist from Bachem) or ddH_2_O as vehicle control. Scratches were photographed at selected time points using an Olympus CK841 microscope (JP) and quantified with ImageJ software [19]. For the transwell assay, 1 × 10^5^ cells were placed into an insert (Millicell, 8 μm pore size; Merck Millipore, MA, USA) with DMEM medium containing 1% FBS and 1 µM opioid, 3.3 nM ruxolitinib (Selleckchem, TX, USA) or ddH_2_O. Inserts were placed into a 24-well plate filled with DMEM medium containing 5% FBS as a chemoattractant. After incubation at 37°C, cells that migrated through the membrane were fixed with 4% formaldehyde and 99% ice-cold methanol, and stained with DAPI (100 ng/mL). Insert membranes were imaged on an Olympus IX71 fluorescence microscope and migrated cells were quantified by ImageJ software.

### *In vivo* metastasis assay

*C.Cg-Rag2^tm1Fwa^ Il2rg^tm1Wji^* (*Rag2^−/−^γc^−/−^*) were backcrossed onto the breast cancer susceptible *Balb/C* background for more than eight generations, and *Balb/C* microsatellite marker analysis with transplant efficiency confirmed isogenicity. Mice were maintained at the University of Veterinary Medicine Vienna under specific pathogen free (SPF) conditions. 1 × 10^6^ MDA-MB-231 were injected into the fourth mammary fat pad of female *Rag2^−/−^γc^−/−^* mice or 1 × 10^4^ 4T1 cells were injected into the fourth mammary fat pad of female wildtype mice. When tumors reached about 100 mm^3^ in volume, the primary tumors were resected. Mice were then randomly divided into vehicle control (PBS) and opioid-treated groups. To mimic human pain management, treatment was started immediately after tumor resection (2 mg/kg opioid intraperitoneal injection; every 24 h). Ten days after primary tumor removal, mice were sacrificed, lungs were perfused, isolated and the lung weight was assessed. Primary tumor volumes were calculated according to the formula: length × (diameter)^2^ × **π**/6.

### Histopathology

Lungs were fixed overnight in 4% Roti-Histofix (Carl Roth, GER), dehydrated, paraffin-embedded and cut in 4 μm sections. Sections were stained with Hematoxylin (Merck, GER) and Eosin G (Carl Roth). For immunohistochemical staining, heat-mediated antigen retrieval was performed in citrate buffer at pH 6.0 (Dako, CA, USA; S1699). Lung sections were then stained with antibodies against cleaved Caspase-3 (dilution 1:200, CST; 9661), Ki-67 (dilution 1:1.000, eBioscience; 14.5698.80), Vimentin (dilution 1:200, CST, 5741) or CK8 (dilution 1:50, Leica microsystems, GER; NCL-L-CK8-TS1) using standard protocols. Images were taken using an Olympus IX71 microscope.

### Oncomine data analyses

Gene expression datasets from human cancer patients were analyzed for the expression levels of *OPRD1, OPRK1* or *OPRM1* by using the Oncomine Platform (Thermo Fisher, Ann Arbor, MI) [20]. Briefly, the *P*value cutoff for statistical significance was set at <0.05, while the fold change cutoff was set to 1.5 and the gene rank cutoff was defined as ‘all’.

### Statistics

Student *t* test, one-way ANOVA and two-way ANOVA test were performed using GraphPad Prism Software version 5.04. The differences in mean values among groups were evaluated and expressed as the mean ± SD. *P* values less than 0.05 were considered statistically significant (* *P* < 0.05; ** *P* < 0.01; *** *P* < 0.001; **** *P*< 0.0001).

### Study approval

All experiments were approved by the institutional animal care committee and review board, and conform to Austrian law (BMBWF-68.205/0094-V/3b/2018).

## Results

### Expression of δ-opioid receptors and association with solid tumor progression

**Fig. 1:**
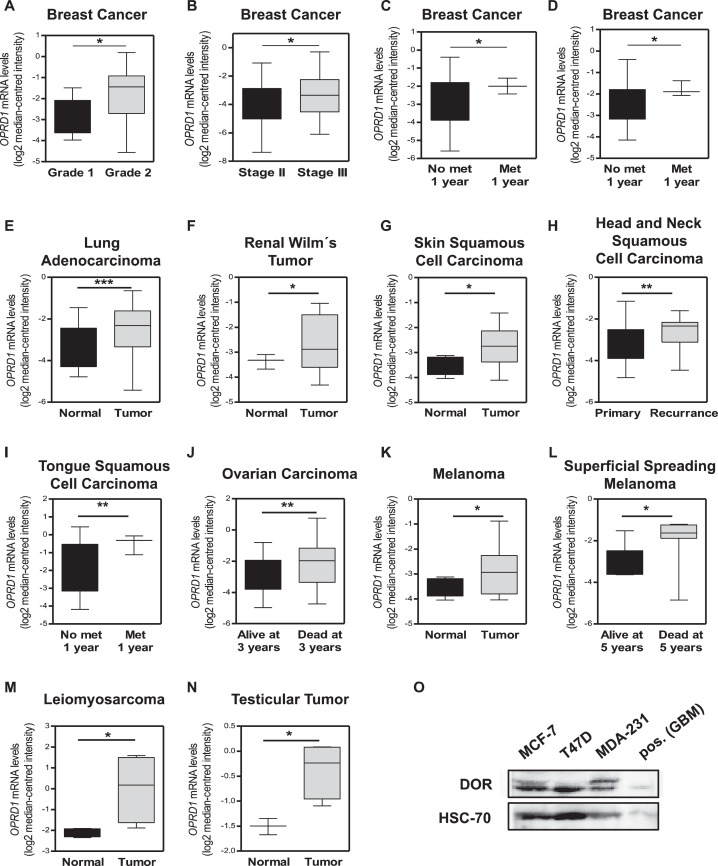
Delta opioid receptor levels are increased in aggressive cancers. (A-N) Data mining of *OPRD1* mRNA expression levels significantly increased in various human cancer patient datasets. All data were extracted from the Oncomine Platform from the following specified studies (indicated in italics; individual *P*-values are indicated by stars within each box plot). (A, B) Box plots showing *OPRD1* mRNA expression levels at different grades (2.2-fold; *P*-value: 0.026; *Bittner Breast)* (A) or stages (1.6-fold; *P*-value: 0.044; *Miyake Breast)* (B) of mammary tumor progression*.* (C, D) Box plots showing *OPRD1* mRNA expression levels in breast cancer patients with or without metastatic events at 1 y (1.8-fold; *P*-value: 0.03; *Desmedt Breast*) (C); (1.7-fold; *P*-value: 0.024; *Minn Breast 2)* (D). (E-N) Box plots showing *OPRD1* mRNA expression in various samples from cancer or normal tissues (as indicated), including lung adenocarcinoma (1.8-fold; *P*-value: 0.001; *Okayama Lung*) (E), renal Wilm´s tumor (1.6-fold; *P*-value: 0.021; *Cutcliff Renal*) (F), squamous cell carcinomas of skin (1.7-fold; *P*-value: 0.021; *Riker Melanoma*) (G), head and neck (1.6-fold; *P*-value: 0.01; *Ginos Head-Neck*) (H) and tongue (2.8-fold; *P*-value: 0.008; *Rickman Head-Neck*) (I), ovarian carcinoma (1.6-fold; *P*-value: 0.001; *Bild Ovarian*) (J), melanoma (1.6-fold; *P*-value: 0.03; *Riker Melanoma*) (K), superficial spreading melanoma (2.4-fold; *P*-value: 0.019; *Xu Melanoma*) (L), leiomyosarcoma (4.4-fold; *P*-value: 0.041; *Quade Uterus*) (M), and testicular tumor (2.7-fold; *P*-value: 0.02; *Skotheim Testis*) (N). (O) δ-opioid receptor (DOR) protein expression in MCF-7, T47D, and MDA-MB-231 cells was determined by Western blotting. Neuronal LN229 cells served as positive control (pos. GBM) for opioid receptor expression. HSC-70 was used as loading control. Blots are representative of 3 independent experiments.

To obtain a comprehensive view of the role of DOR in breast cancer disease progression and metastasis, gene expression analyses were performed using breast cancer patient datasets publicly available from the Oncomine database. As shown in Fig. 1A, the expression of DOR (*OPRD1*) mRNA was significantly higher in Grade 2 than in Grade 1 mammary malignancies. Moreover, Stage III breast cancer exhibited higher DOR expression than Stage II tumors (Fig. 1B). Notably, expression of DOR mRNA correlated with enhanced disease progression in breast cancer metastasis (Fig. 1C and D).

Interestingly, these observations were not restricted to mammary malignancies. Oncomine data evaluation also revealed significant associations of increased DOR expression in other epithelial cancer subtypes including lung adenocarcinoma, renal cell cancer, and skin squamous cell carcinoma, compared with healthy tissues (Fig. 1E-G). Furthermore, data mining revealed profound associations of increased DOR expression and cancer recurrence or survival in squamous cell carcinomas of head and neck, tongue or ovarian cancer (Fig. 1H-J). In addition, increased expression of DOR was found in cutaneous melanoma compared with healthy skin samples, and higher DOR levels were associated with shorter survival of superficial spreading melanoma patients (Fig. 1K and L). Tumors of mesenchymal or germ cell origin were also associated with increased DOR expression, including leiomyosarcoma and cancer of testis versus healthy control tissues (Fig. 1M and N).

In addition to higher DOR expression, data mining revealed that various aggressive cancer types also overexpress κ- (KOR, *OPRK1*) and µ- (MOR, *OPRM1*) opioid receptors. KORs were found to be increased at the mRNA level in adenocarcinomas of the lung and pancreas, prostate carcinoma and myxoid/round cell liposarcoma, versus healthy control tissues (Fig. S1A-D). Expression of MOR mRNA was significantly upregulated in clear cell renal cell carcinoma and pancreatic ductal adenocarcinoma versus healthy tissue (Fig. S1E-F), and increased MOR levels were also associated with patient survival/tumor recurrence in squamous cell lung carcinoma, esophageal squamous cell carcinoma, and colon adenocarcinoma (Fig. S1G-I). Thus, these broad data mining analyses indicate that opioid receptors are more highly expressed in various human solid cancers, and particularly elevated DOR expression levels are associated with tumor progression and accelerated metastatic disease.

### Opioid treatment with a DOR agonist increases migration/metastasis of breast cancer cells

**Fig. 2:**
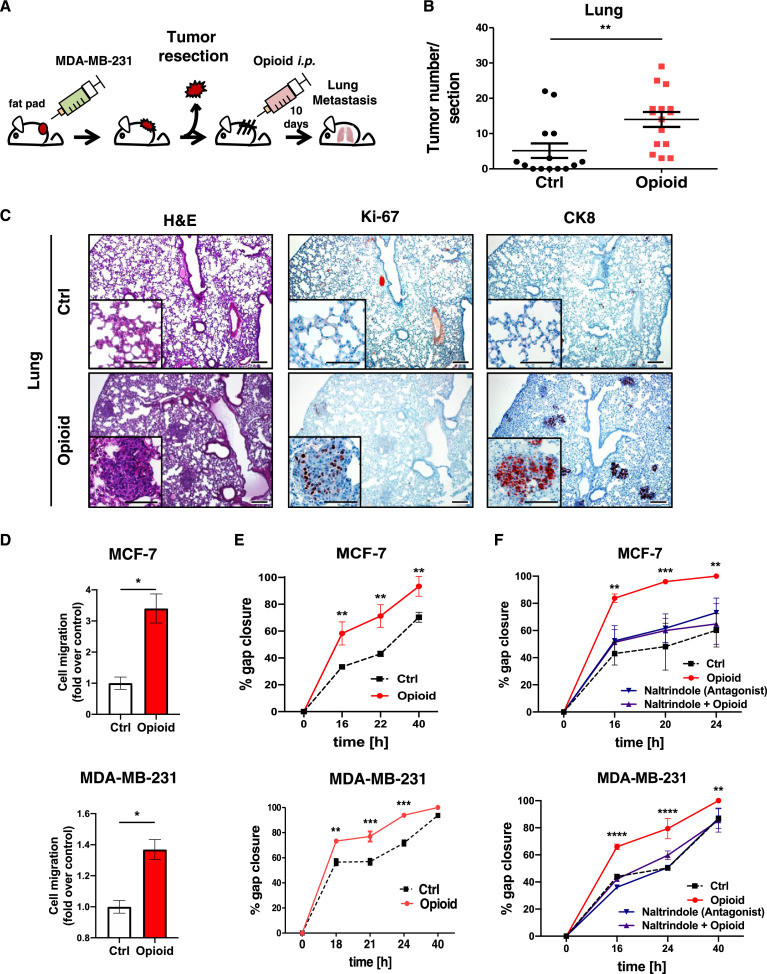
A DOR agonist increases metastasis/migration in breast cancer cells. (A) Experimental scheme. (B) Quantification of metastasis in the lungs of opioid- and saline-treated *Rag2^−/−^γc^−/−^* mice. Graphs represent quantification of CK8-positive tumors per section as mean ± SD (*n* = 14 for PBS-treated mice and *n* = 15 for opioid-treated mice; magnification of section: 40×; **P < 0.01; Student *t* test). (C) Immunostainings of lungs from control and opioid-treated mice for H&E, Ki-67 and CK8 (magnification: 40× and 100×; scale bars 200 µm and inserts 100 µm). (D) Transwell migration assay of MCF-7 (upper panel; *P < 0.05) and MDA-MB-231 cells (lower panel; *P < 0.05) after opioid stimulation (1 µM, 16 h incubation). Controls (ctrl) were treated with ddH_2_O (Bar graphs represent the fold change of migrated cells over the control as mean ± SD in duplicates from a single experiment, representative of 3 independent experiments; unpaired two-sided Student *t* test). (E) Scratch assays of MCF-7 and MDA-MB-231 cells exposed to opioid (1 µM) or ddH2O (ctrl). Quantification represents gap closure (migration) rates as mean ± SD of the % of the closure of original gap of duplicates from a single experiment, representative of three independent experiments (MCF-7: **P < 0.01 versus control; MDA-MB-231: **P < 0.01 versus control; ***P < 0.001 versus control; two-way ANOVA). (F) Scratch assay of MCF-7 and MDA-MB-231 cells treated with 1 µM opioid in the absence or presence of 10 µM naltrindole. Quantification represents gap closure (migration) rates as mean ± SD of the % of the closure of original gap of duplicates from a single experiment, representative of 3 independent experiments (MCF-7: **P < 0.01 versus control; ***P < 0.001 versus control; MDA-MB-231: **P < 0.01 versus control; ****P < 0.0001 versus control; two-way ANOVA). DOR, δ-opioid receptors

Opioids used as common analgesics bind to and activate opioid receptors, including DOR. The striking data on elevated DOR mRNA levels and breast cancer progression as well as the clinical need to better understand mechanisms of breast cancer progression prompted us to further explore the role of DOR in opioid-induced breast cancer metastasis. To establish model cell systems for *in vitro* and *in vivo* experiments, three human breast cancer cell line models of epithelial (MCF-7 and T47D) or mesenchymal origin (MDA-MB-231) were examined for opioid receptor expression. All three cell lines were found to endogenously express DOR protein (Fig. 1O). Next, to investigate opioid-induced breast cancer metastasis, we used a spontaneous metastasis breast cancer model that mimics human breast cancer patients [21] whereby MDA-MB-231 cells were implanted into mammary fat pads of fully immunocompromised *Rag2^−/−^γc^−/−^* mice. After surgical removal of the primary tumor, mice were treated with a classic DOR agonist [D-Ala^2^,D-Leu^5^]-Enkephalin (hereby indicated as “opioid”) for 10 d (Fig. 2A), and distant metastases were examined in the lungs of the mice by immunohistochemistry (Fig. 2B and C). The primary tumor removal prolongs the life span of the tumor-bearing mice, but does not prevent the tumor from metastasizing to distant parts of the mice. Compared to the vehicle control, lungs of opioid-treated mice showed more clusters of Ki-67 and Cytokeratin 8 (CK8) positive cells (Fig. 2C), identifying them as metastatic breast cancer cells [22]. To evaluate immune cell contribution, we next employed an immunocompetent allograft breast cancer model. We performed opioid treatment in Balb/C wildtype mice bearing murine 4T1 breast cancer cell orthotopic transplants, which also resulted in significantly more metastatic lung nodules than vehicle controls (Fig. S2A). These 2 orthotopic transplant models demonstrate that opioid treatment significantly increased lung metastasis of primary breast cancer tumor cells irrespective of immune cell contribution.

Next, migration of DOR-expressing breast cancer cells was examined. Transwell and scratch assays showed that exposure of epithelial MCF-7 and T47D, and mesenchymal MDA-MB-231 breast cancer cells to opioid significantly enhanced migration (Fig. 2D, and Fig. S2B, 2E and S2C). Opioid-induced migration was abolished by naltrindole, a DOR-selective antagonist (Fig. 2F and Fig. S2D). Importantly, opioid exposure affected neither proliferation nor cell cycle distribution (Fig. S2E, S2F). Our findings indicate that opioids promote breast cancer cell migration via DOR stimulation.

### STAT3 is activated by a DOR agonist in breast cancer cells

**Fig. 3:**
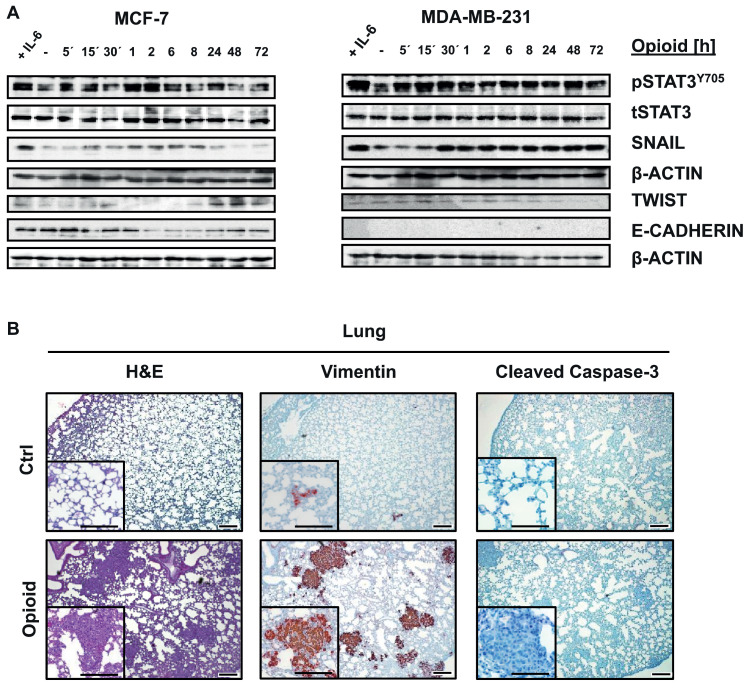
Opioid exposure induces STAT3 tyrosine phosphorylation and expression of individual EMT-related markers in breast cancer cells. (A) MCF-7 and MDA-MB-231 cells were treated with 1 µM opioid for 5 min – 72 h and analyzed for pSTAT3^Y705^, tSTAT3, SNAIL, TWIST and E-Cadherin levels by immunoblotting. β-ACTIN was used as loading control. Blots are representative of 3 independent experiments. (B) Representative H&E, Vimentin and Cleaved Caspase-3 stained lung sections of mice engrafted with MDA-MB-231 cells after primary tumor removal and postsurgical opioid treatment for 10 d (original magnification: 40× and 100×; scale bars 200 µm and inserts 100 µm).

Migration of epithelial carcinoma cells can be promoted through multiple core cancer pathways, including the JAK1/2-STAT3/5, β-Catenin/WNT, RAS/RAF-ERK1/2 or TGF-β/SMAD2/3 pathways. Whereas incubation of MCF-7, T47D and MDA-MB-231 cells with opioid for different time periods had no significant effect on activation of β-Catenin (ABC), ERK1/2 or SMAD2/3 (Fig. S3A), immunoblotting showed that cell exposure to opioid resulted in a rapid increase in STAT3 activation (pYSTAT3) (Fig. 3A and Fig. S3B). Many cytokine and growth factor receptors trigger JAK1/2-STAT3/5 activation, however, it was somewhat unexpected and striking that a GPCR such as DOR could also activate STAT3 in breast cancer cells to a similar extent as IL-6 cytokine stimulation (Fig. 3A). In contrast, STAT5A/B transcription factors also known to have oncogenic functions and promote breast cancer progression remained unaffected by DOR activation (Fig. S3C). Oncomine data analyses also identified an increase in DOR mRNA expression correlating with increased *in vitro* sensitivity to the tyrosine kinase inhibitor dasatinib in breast cancer cell lines (Fig. S3D). Given that dasatinib can inhibit the activity of various tyrosine kinases including JAKs, these data also suggest that increased DOR expression results in dependence on signaling pathways targeted by dasatinib, such as JAK-STAT.

STAT3 can control cell migration through promotion of EMT [23]. To test whether opioid treatment can induce EMT, breast cancer cells were analyzed for classical EMT-related transcription factors controlled by STAT3, in particular the E-Cadherin repressors *SNAIL* and *TWIST* [24], [25], [26]. Immunoblotting revealed that opioid treatment led to an upregulation of SNAIL in all tested breast cancer cells (Fig. 3A and Fig. S3E, S3F and S3G). Moreover, TWIST was enhanced in opioid-treated MCF-7 and T47D cells. Consistently, E-Cadherin was decreased in MCF-7 and T47D cells. Immunostainings of lungs from opioid-treated mice bearing breast cancer xenografts further demonstrated that the observed metastases are highly positive for Vimentin, a further mesenchymal marker, and negative for cleaved Caspase-3 (Fig. 3B). Therefore, our data suggest that opioids may promote EMT through STAT3 activation, loss of E-Cadherin and gained Vimentin expression.

### Role of STAT3 in opioid-mediated cell migration and metastasis

**Fig. 4:**
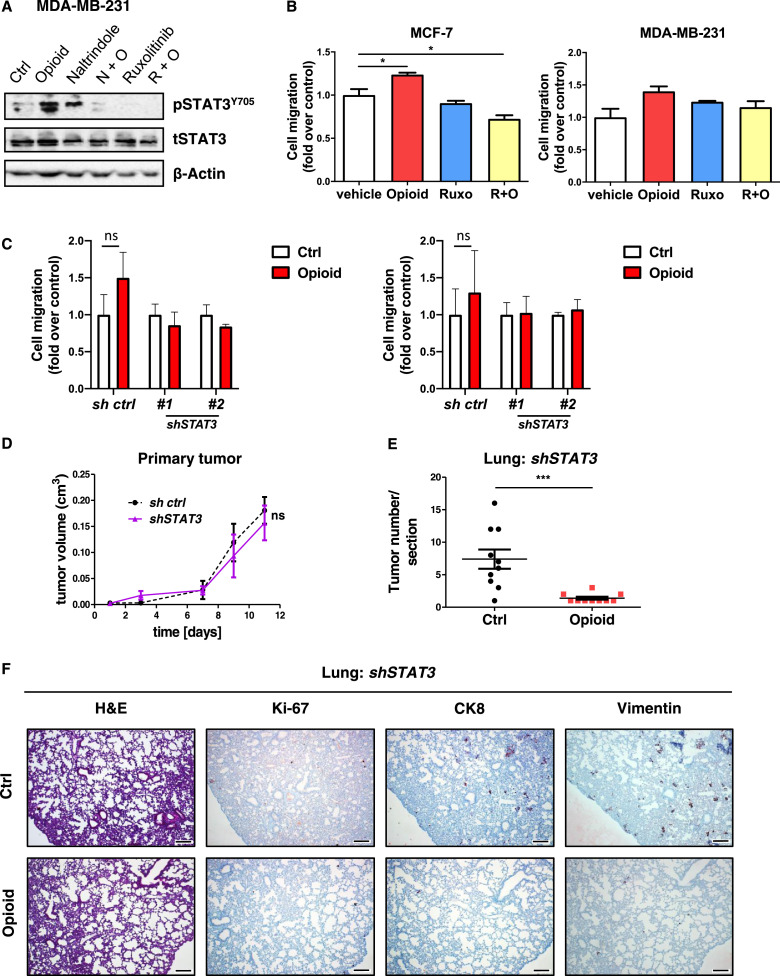
Inhibition of STAT3 attenuates opioid-triggered breast cancer cell migration and metastasis. (A) MDA-MB-231 cells were treated with 1 µM opioid in the absence or presence of naltrindole (10 µM) or ruxolitinib (3.3 nM), and analyzed for pSTAT3^Y705^ and tSTAT3 by immunoblotting. β-ACTIN was used as loading control. (B) Transwell assay. MCF-7 and MDA-MB-231 cells were exposed to opioid (1 µM) alone or together with ruxolitinib (3.3 nM). After 16 h, cells that migrated through the membrane pores were quantified (Bar graphs represent the fold change of migrated cells over the control as mean ± SD in duplicates from two independent experiments; *P < 0.05 opioid versus vehicle, one-way ANOVA). (C) MCF-7 and MDA-MB-231 cells were infected with unrelated *control-shRNA* (*sh ctrl*), *STAT3-shRNA* #1, or *STAT3-shRNA* #2, and examined for migration activity after opioid treatment by transwell assay. Controls were treated with ddH_2_O (Bar graphs represent the fold change of migrated cells over the control as mean ± SD in duplicates from 2 independent experiments; not statistically significant (ns), unpaired two-sided Student *t* test). (D) Volumes of primary tumors formed by MDA-MB-231 cells infected with *sh ctrl* or *STAT3-shRNA* (*shSTAT3*) over time (*n* = 10 for PBS-treated mice and *n* = 10 for opioid-treated mice; not statistically significant (ns), two-way ANOVA). (E) Quantification of tumor metastasis in the lungs of opioid-treated *Rag2^−/−^γc^−/−^* mice engrafted with *STAT3-shRNA* infected MDA-MB-231 cells. Graphs depict CK8-positive tumor spots per section as mean ± SD (*n* = 10 for PBS-treated mice and *n* = 10 for opioid-treated mice; magnification of section: 40×, ***P < 0.001, unpaired two-sided Student *t* test). (F) Representative images of H&E, Ki-67, CK8 and Vimentin immunostainings of lungs from mice engrafted with *STAT3*-silenced MDA-MB-231 cells after primary tumor removal and postsurgical opioid treatment for 10 d (original magnification: 40×; scale bars 200 µm). Control mice were treated with PBS (vehicle).

Next, the role of STAT3 activation in opioid-mediated cell migration was examined. First, cell motility was assessed in the presence of the FDA-approved JAK1/2 inhibitor ruxolitinib. Ruxolitinib blocked opioid-induced STAT3 activation (Fig. 4A) and prevented opioid-triggered migration (Fig. 4B and Fig. S4A). Opioid-induced STAT3 activation was also reduced by the DOR antagonist naltrindole (Fig. 4A). In a second approach, STAT3 function was blocked by stable RNA knockdown. Infection of breast cancer cells with *STAT3-shRNA* reduced STAT3 expression by more than 70% at both the mRNA and protein level (Fig. S4B, S4C). Importantly, *STAT3* knockdown did not affect cell proliferation, cell cycle distribution or basal migration activity (Fig. S4D-F), but notably it prevented increased migration of breast cancer cells in response to opioid exposure (Fig. 4C and Fig. S4G).

To assess the role of STAT3 in opioid-driven metastasis, MDA-MB-231 cells stably transduced with *STAT3-shRNA* or *control-shRNA* were orthotopically injected into *Rag2^−/−^γc^−/−^* female recipients. Primary tumors formed by *STAT3*-silenced cells were similar in growth kinetics and volume compared to MDA-MB-231 *control-shRNA* cells (Fig. 4D). Following primary tumor resection, mice were treated with opioid or vehicle and lung metastasis was examined. Strikingly and in line with *in vitro* results, animals injected with *STAT3-shRNA* infected cells had significantly less CK8-positive metastatic lesions in the lungs after opioid treatment than vehicle-treated controls (Fig. 4E and F). Lungs from opioid-treated mice injected with *STAT3*-silenced cells also displayed significantly less Ki-67 and Vimentin staining than saline-injected controls (Fig. 4F). Taken together, we show here for the first time that DOR-triggered migration and metastasis of breast cancer cells acts via oncogenic JAK1/2-STAT3 signaling, promoting breast cancer progression potentially through increased EMT.

## Discussion

Opioid usage in cancer patients is currently under scrutiny as evidence is emerging of adverse effects on tumor progression. Concerningly, opioids are taken as painkillers to alleviate chronic disease symptoms, which has led to an ongoing debate over opioid safety and the rise of an “opioid crisis”. In this study, we provide important mechanistic insights into the detrimental biological functions of opioids in breast cancer progression, as evidenced by their capacity to increase migration and metastasis through STAT3 activation and EMT reprograming.

We mined public gene expression datasets that revealed DOR expression to be upregulated in many solid cancers, focusing further on advanced and invasive breast cancer, where DOR expression levels correlated with accelerated metastasis and disease progression. Previous studies revealed that signaling of DOR strongly differs from signaling of μ- and κ-opioid receptors (MOR, KOR). This results in differences in the regulation of nociception and emotional responses [27], but also proliferation, differentiation and survival of cells outside the central nervous system [28,29]. It is unclear what mediates the specificity of DOR functions, but alternative G protein coupling may be relevant. In contrast to MOR and KOR, DOR activates G_q/11_ proteins [30] and these were found to promote metastasis [31].

Using *in vitro* techniques and different breast cancer transplant models in immunosuppressed and immunocompetent mice, we demonstrated that DOR activity accelerates breast cancer migration and metastasis irrespective of immune cell status. Furthermore, our pharmacologic and genetic interference studies culminate on an essential role of JAK1/2-STAT3 activation in opioid-induced EMT processes through reduced E-Cadherin expression. Whether this mechanism involves indirect or direct JAK1/2-STAT3 pathway signaling still needs to be further examined, however, the rapid induction of pY705-STAT3 activating phosphorylation upon DOR ligation suggests that a direct mechanism may be likely. Indeed, JAK1/2 activation by GPCRs was previously reported to be facilitated by a direct interaction with the α subunit of G_q_ proteins [32]. DORs are coupled to G_q_ proteins, likely contributing to JAK1/2-STAT3 pathway activation. Furthermore, signaling through GPCRs such as chemokine or cardiotrophin receptors were previously shown to trigger significant pYSTAT3 levels [33]. Interestingly, STAT5 activity remained unaffected by opioid treatment despite being a downstream target of JAK1/2. This observation could be due to cell type-specific expression level differences, where higher STAT3 levels may contribute to the dominant JAK1/2-STAT3 signaling mediated by DOR activation in breast cancer cell migration and metastasis.

An increase in pY705-STAT3 upon DOR stimulation has not been observed formerly. SNC80, a DOR agonist, was reported to increase pS727-STAT3 in murine mesenchymal stem cells [29], which is important for the mitochondrial activity of STAT3 [34]. However, the direct role of DOR activation by SNC80 in this study remained unclear and so the induction of pS727-STAT3 is likely to represent an off-target effect of this agonist [35]. In contrast, stimulation of pY705-STAT3 was reported for opioid receptor like-1 overexpression in transfected human embryonic kidney (HEK293) cells [36]. In this artificial cell system, opioid receptor like-1 activated STAT3 via transfected G_α16_, whose expression is usually restricted to hematopoietic cells and is not significantly expressed in epithelial cells [37]. Our data thus show for the first time that stimulation of DORs endogenously expressed in breast cancer cells rapidly and strongly induces pY705-STAT3 and downstream gene transcription.

Previous studies revealed that β-endorphin and other selective DOR agonists inhibit thymic and splenic T cell proliferation and cytokine production [38], [39], [40], suggesting that DOR signaling could promote metastasis by suppressing immune function. However, breast cancer metastasis was enhanced in both immunocompetent and immunodeficient mouse models, implying that the immunosuppressive effect of opioids is not a key driver for breast cancer progression.

We demonstrated that DOR stimulation enhances breast cancer cell migration, suggesting that opioid-promoted metastasis originates from enhanced cancer cell motility. DOR-stimulated cell migration has been previously observed for non-tumor cells such as epithelial cells, fibroblasts and keratinocytes [28,41,42]. Importantly, we found that opioid exposure failed to induce classical migration pathways such as MAPK/ERK, TGF-β/SMAD2/3 or WNT/β-Catenin in breast cancer cells, but activated the migratory JAK1/2–STAT3 axis. Opioid-induced STAT3 activation without any concomitant effect on the WNT/β-Catenin pathway was surprising, as ABC/WNT signaling is considered an essential driver of cell motility and it synergizes in colorectal carcinoma progression with oncogenic STAT3 activation [43]. Since WNT and STAT3 share common downstream target genes, such as *D-type cyclins* and *c-MYC*, we conclude that activation of both pathways is not required for opioid-mediated breast cancer migration and metastasis.

The observed lung metastases in opioid-treated xenografts were all found to be Vimentin positive. Vimentin has a critical role in metastasis by stabilizing mature invadopodia, which is a prerequisite for invasive spread of cancer cells. Vimentin is expressed in MDA-MB-231 cells [44], but we found even more intense Vimentin expression in MDA-MB-231 cells after opioid treatment in transplanted tumors. Enhanced Vimentin expression in MDA-MB-231 cells after opioid exposure could be a downstream effect of further increased STAT3 activation, as STAT3 can bind to and activate the Vimentin promoter [45]. Opioid exposure also resulted in a consistent upregulation of the EMT marker SNAIL and downregulation of E-Cadherin in breast cancer cells. SNAIL enhances cell movement via RhoB upregulation, which alters focal adhesion dynamics [46]. The loss of E-Cadherin expression is striking and could be a combined consequence of upregulated E-Cadherin repressor SNAIL as well as STAT3 activation. In contrast, the loss of E-Cadherin after breast cancer cells were exposed to opioid for 1 h suggests a rapid internalization of the transmembrane glycoprotein after DOR stimulation. E-Cadherin is internalized via Clathrin-coated pits, which are also involved in opioid-induced endocytosis of DORs [47]. Stimulated DORs are maximally internalized within 1 h [48], thus a co-internalization via Clathrin-coated pits might account for the observed loss of E-Cadherin in opioid-exposed breast cancer cells.

Several clinically relevant opioids such as morphine, methadone, and fentanyl analogues may also trigger DOR signaling [49,50]. Therefore, application of these potent analgesics in cancer patients or aged patients with enhanced cancer risk has to be considered with more care, increased risk assessment and further mechanistic studies. We do not propose STAT3 or JAK1/2 inhibition as promising therapies to be combined with opioids, as this could result in severe immunosuppression or altered stromal-tumor cell signaling [51]. Moreover, drugs like ruxolitinib are known to alter cancer cell metabolism and may increase the risk of breast cancer by alternative pathways [51].

In conclusion, we show here for the first time that DOR triggered migration and metastasis of breast cancer cells, which acts via oncogenic JAK1/2-STAT3 signaling and potentially promotes breast cancer progression through increased EMT. Thus, our findings emphasize the importance of selective and restricted use of opioid-based pain medication in cancer patients or elder patients at high-risk. We advocate the need for safer pain medication that does not activate oncogenic pathways such as JAK1/2-STAT3 signaling. Thus, further mechanistic studies will be important and required to identify safer pain medication alternatives.

## Author contribution

D.A.F. and S.T. designed and supervised the study, S.T., V.M.K., and D.P.E. performed experiments. S.T., H.A.N. and D.P.E. analyzed data. F.A., D.A.F. and R.M. provided reagents and analytic tools. S.T., D.A.F., H.A.N. and R.M. wrote the manuscript.

## Financial support

D.A.F. was supported by an Austrian Science Fund (FWF) grant P27248-B28. R.M. was supported by the FWF grants SFB-F04707, SFB-F06105, and under the frame of ERA-NET (I 4157-B). H.A.N. was supported by the FWF, under the frame of ERA PerMed (I 4218-B). R.M. and H.A.N. were also generously supported by a private donation from Liechtenstein. F.A. was supported by FWF grants W1213, P25629, the priority program ACBN of the University of Salzburg, the smart specialization center Cancer Cluster Salzburg and the Biomed Center Salzburg (grants 20102-P1601064-FPR01-2017 and 20102-P1901165-KZP by the County of Salzburg).

## Conflict of interest

All authors declare that they have no competing financial or nonfinancial interests that might have influenced the performance or presentation of the work described in this manuscript.

## Appendix Supplementary materials

Image, application 1
