# Supplementary material for: Opioids drive breast cancer metastasis through the δ-opioid receptor and oncogenic STAT3
Source: Neoplasia. 2021 Jan 16;23(2):270–9. doi: 10.1016/j.neo.2020.12.011 (PMC7815495; doi:10.1016/j.neo.2020.12.011)
Supplement: Supplementary file 1 [file mmc1.docx]

**Tripolt *et al.* Supplemental Figures**

**Supplemental Figure S1. Additional Oncomine gene expression data for k- and µ-opioid receptor expression in various solid tumors.** (**A-D**) Comparison of *OPRK1* mRNA expression in various cancers compared with normal healthy tissues, including lung adenocarcinoma (1.5-fold; P-value: 0.004; *Okayama Lung*), pancreatic adenocarcinoma (1.7-fold; P-value: 0.005; *Iacobuzio-Donahue Pancreas 2*)*,* prostate carcinoma (3.5-fold, P-value: 4.7E-4; *Welsh Prostate*)*,* and myxoid/round cell liposarcoma (2-fold; P-value: 2.09E-9; *Barretina Sarcoma*)*.* (**E-I**) Comparison of *OPRM1* mRNA expression in various normal or cancer tissues, including renal carcinoma (2.6-fold; P-value: 7.5E-15; *Jones Renal*)*,* pancreatic ductal adenocarcinoma (1.6-fold; P-value: 0.002; *Buchholz Pancreas*), squamous cell lung carcinoma (2.0-fold; P-value: 0.008; *Bild Lung*), esophageal squamous cell carcinoma (4.8-fold; P-value: 1.9E-4; *Aoyagi Esophagus*), and colon adenocarcinoma (1.8-fold; P-value: 0.004; *Jorissen Colorectal 3*).

**Supplemental Figure S2. Opioid-treatment does not affect proliferation. (A)** Quantification of tumor metastases on the lung surface of *Balb/C* wildtype mice injected with 4T1 cells and treated with opioid for ten days after primary tumor resection. Controls (ctrl) were treated with PBS (n=4 for PBS-treated mice, n=6 for opioid-treated mice; *P<0.05; data are presented as the mean ± SD, unpaired two-sided Student´t t-test). **(B)** T47D cells were exposed to opioid for 48 h and examined for migration activity by transwell assay. Controls (ctrl) were treated with ddH_2_O (*P<0.05 versus control; bar graphs represent the fold change of migrated cells over the control as mean ± SD in duplicates from a single experiment, representative of three independent experiments; unpaired two-sided Student´s t-test). **(C)** T47D cells were incubated with 1 µM opioid and tested for cell migration by scratch assay. Quantification represents gap closure (migration) rates as mean ± SD of the % of the closure of original gap of duplicates from a single experiment, representative of three independent experiments (****P<0.0001 versus control; two-way ANOVA). **(D)** T47D cells were treated with 1 µM opioid in the absence or presence of 10 µM naltrindole and examined for motility by scratch assay. Quantification represents gap closure (migration) rates as mean ± SD of the % of the closure of original gap of duplicates from a single experiment, representative of three independent experiments (not statistically significant; two-way ANOVA**)**. **(E)** Growth curves. MCF-7, T47D and MDA-MB-231 cells were incubated with 1 µM opioid and counted by FACS every 24 h. **(F)** Cell cycle analysis. MCF-7, T47D and MDA-MB-231 cells were treated with 1 µM opioid for 24 h and examined for cell cycle distribution by FACS. Representative histograms of two independent experiments show the percentages of cells in G1/G0, S, G2/M and subG0 phase.

**Supplemental Figure S3. Opioid-induced signaling. (A)** MCF-7, T47D and MDA-MB-231 cells were treated with opioid for indicated time periods and examined for ABC, pp42/p44^T202/Y204^, tp42/p44, pSMAD2^S465/467^/SMAD3^S423/425^ and tSMAD2/3 by Western blotting. Cells exposed to rhEGF- (5 min; 100 ng/ml) or rhTGF- (40 min; 1 ng/ml) were used as positive controls. GAPDH or HSC70 served as loading control. Blots are representative of 3 independent experiments. **(B)** T47D cells were exposed to 1 µM opioid for 5-60 min, and probed for pSTAT3^Y705^ and total STAT3 by immunoblotting. β-ACTIN was used as loading control. Blot is representative of 3 independent experiments. **(C)** Western blot analysis of opioid-treated MCF-7, T47D and MDA-MB-231 cells for pSTAT5^Y694^ and tSTAT5. Cells incubated with rhIL-2 (5 min; 100 ng/ ml) served as positive pSTAT5 control. β-ACTIN was used as loading control. Blots are representative of 3 independent experiments. **(D)** Box plot showing a significant (P-value: 0.026; *Huang CellLine*) 1.8-fold increase in *OPRD1* mRNA expression levels in BCa cell lines sensitive to dasatinib compared to those resistant to dasatinib. Data were extracted from the Oncomine™ platform. (**E**) T47D cells were treated with 1 µM opioid for 5 min – 72 h, and assessed for SNAIL, TWIST and E-Cadherin by immunoblotting. β-ACTIN and HSC-70 were used as loading controls. Blots are representative of 3 independent experiments. (**F**) Densitometric analysis of protein expression of STAT3 phosphorylation normalized to the corresponding tSTAT3 protein levels measured by Western blot analysis. (**G**) Densitometric analysis of protein expression of proteins SNAIL, TWIST, E-Cadherin and tSTAT3 normalized to β-ACTIN as measured by Western blot analysis.

**Supplemental Figure S4. Drug- and shRNA-induced STAT3 inhibition in breast cancer cells. (A)** Transwell assay. T47D cells were exposed to opioid (1 µM) in the absence or presence of 3.3 nM ruxolitinib. Controls were treated with DMSO (Bar graphs represent the fold change of migrated cells over the control as mean ± SD in duplicates from two independent experiments; **P<0.01 opioid versus vehicle, one-way ANOVA). **(B)** MCF-7, T47D and MDA-MB-231 cells were infected with *control-shRNA* (*sh ctrl*), *STAT3-shRNA* #1 or *STAT3-shRNA* #2 and assessed for *STAT3* mRNA levels by qPCR. **(C)** MCF-7, T47D and MDA-MB-231 cells infected with control-shRNA (*sh ctrl*), *STAT3-shRNA* #1 or *STAT3-shRNA* #2 were analyzed for STAT3 protein expression by immunoblotting. β-ACTIN was used as loading control. **(D)** Growth curves. MCF-7, T47D and MDA-MB-231 cells infected with *control-shRNA* (*sh ctrl*), *STAT3-shRNA* #1 or *STAT3-shRNA* #2 were counted by FACS every 24 h (graphs represent the average of two independent experiments ± SD, not statistically significant (ns), two-way ANOVA). **(E)** Cell cycle analysis. STAT3-silenced MCF-7, T47D and MDA-MB-231 cells were examined for cell cycle distribution by FACS. Bar graphs showing cells in G1/G0, S, G2/M and subG0 phase. Data are presented as the mean values from two independent experiments ± SD. **(F)** Scratch assays were performed to compare migration activity of T47D, MCF-7 and MDA-MB-231 cells infected with *control-shRNA*, *STAT3-shRNA* #1 or *STAT3-shRNA* #2 under basal conditions (graphs represent the average of two independent experiments ± SD, ns, two-way ANOVA). **(G)** T47D cells infected with control-shRNA (*sh ctrl*) or *STAT3-shRNA* were examined for migration activity after exposure to 1 µM opioid for 48 h (Bar graphs represent the fold change of migrated cells over the control as mean ± SD in duplicates from two independent experiments; not statistically significant, unpaired two-sided Student´s t-test).
